# Impact of needle positioning on ablation success of irreversible electroporation: a unicentric retrospective analysis

**DOI:** 10.1038/s41598-020-78660-0

**Published:** 2020-12-14

**Authors:** René Michael Mathy, Parham Tinoush, Ricardo Daniel da Florencia, Alexander Braun, Omid Ghamarnejad, Boris Radeleff, Hans-Ulrich Kauczor, De-Hua Chang

**Affiliations:** Clinic for Diagnostic and Interventional Radiology, Heidelberg University Hospital, Im Neuenheimer Feld 420, 69120 Heidelberg, Germany

**Keywords:** Cancer imaging, Cancer therapy, Liver cirrhosis, Hepatocellular carcinoma, Outcomes research

## Abstract

Irreversible electroporation (IRE) is an ablation procedure in which cell death is induced by ultrashort electrical pulses. In this unicentric retrospective study we investigated the influence of needle positioning on ablation success. 15 IREs with residual tumor after ablation, detected in the first follow-up MRI, were included, and compared with 30 successful ablations. Evaluation of needle geometry revealed significantly higher values for needle divergence (NDiv, 7.0° vs. 3.7°, *p* = 0.02), tumor-center-to-ablation-center distance (TACD, 11.6 vs. 3.2 mm, *p* < 0.001), tumor-to-needle distance (4.7 vs. 1.9 mm, *p* = 0.04), and tumor diameter per needle (7.5 vs. 5.9 mm/needle, *p* = 0.01) in patients with residual tumor. The average number of needles used was higher in the group without residual tumor after ablation (3.1 vs. 2.4, *p* = 0.04). In many cases with residual tumor, needle depth was too short (2.1 vs. 6.8 mm tumor overlap beyond the most proximal needle tip, *p* < 0.01). The use of a stereotactic navigation system in 10 cases resulted in a lower NDiv value (2.1° vs. 5.6°, *p* < 0.01). Thus, correct needle placement seems to be a crucial factor for success and the assistance of a stereotactic navigation system might be helpful. As most important geometrical parameter TACD could be identified. Main reasons for high TACD were insufficient needle depth and a lesion location out of the needle plane.

## Introduction

Irreversible electroporation (IRE) is a relatively new local ablation method, introduced in 2007 to the US market and used in our department since 2012.

In contrast to other ablative methods, such as microwave ablation or radiofrequency ablation, which rely on thermal effects that lead to necrosis ^1,2^, IRE produces ultra-short electrical pulses that create nanopores in the cell membrane and lead to cell membrane disruption ^3,4^. These nanopores must be permanent and lethal so that the cell cannot recover, which is only achieved if the electric field is strong enough ^5^. In this case cell death occurs mainly because of apoptosis, but to a certain extent also by necrosis, and only in close proximity to the electrodes ^6^. The advantages of this method over thermal ablation techniques are therefore the protection of connective tissue and vessels as well as the avoidance of heat-sink effects, which is caused in thermal ablation techniques by heat dissipation due to blood flow ^7,8^. Thus, IRE is mainly used for nonresectable liver tumors in the vicinity of larger vessels or other heat-sensitive structures. One of the disadvantages of this method is that at least 2 and up to 7 electrodes are placed in or around the tumor, which is more time-consuming and can lead to errors in needle placement ^9^. Moreover, a porcine animal model ^6^ and mathematical models ^10^ have shown that these needles must be placed precisely and in parallel to generate a sufficiently strong electrical field ^11,12^. A needle spacing between 15 and 20 mm is ideal ^5,6,13^. On the one hand, angulation of the electrodes can lead to overcurrent at the location where the electrodes are in close proximity resulting in unwanted joule heating, and on the other hand, incomplete ablation can occur at the locations where the electrodes are further apart ^10,11^. Due to sensitive tissue structures or other anatomical considerations, it is possible that the needle depth of the inserted electrodes is not the same. On the one hand this leads to a distorted ablation area and on the other hand to an underestimation of the actual electrode spacing with a resulting overestimation of the ablation area ^11^. These factors, which are relatively specific to the IRE possibly result in a lower technical success rate in comparison to other ablative techniques ^6,11^. Especially with tumors that are difficult to visualize or with difficult access routes, needle positioning can be very difficult. To improve needle placement, the assistance of stereotactic navigation systems can be used. Using such a navigation system in IRE, both retrospectively ^14^ and prospectively ^9^ it was shown that the intervention time was shorter, the radiation exposure lower and the procedural accuracy, measured as needle deviation from a reference electrode, higher. Several retrospective case series have evaluated the technical ablation success of IRE ^15–18^, but to our knowledge, no study has evaluated in detail the potential reasons for needle placement failure.

In summary, method-inherent, unfavorable needle positioning in IRE is a major reason for incomplete tumor ablation. Due to the use of multiple needles, different geometric scenarios of incorrect positioning are conceivable. The aim of the study is to identify the most relevant faulty arrangements by a retrospective review of patient data from our institution and a head-to-head comparison of interventions with and without complete ablation. Based on the results, thresholds of needle mispositioning should be established for clinical routine.

## Methods

For this retrospective analysis, we identified 15 ablations with residual tumor after IRE of hepatocellular carcinoma (HCC) in our tertiary care hospital between 2013 and 2018. Thirty, randomly selected, technically successful ablations (i.e., no residual tumor) after IRE during the same time period were used for comparison.

Technical success was defined as the absence of a residual tumor on the first follow-up magnetic resonance imaging scan after 6 weeks. Residual tumor was diagnosed if there was a persistent arterial contrast enhancement at the ablation site and washout in the portalvenous phase suggestive of HCC. Ablations without follow-up examination, or with inconclusive findings in follow-up, were excluded.

In total, 45 ablations in 35 patients were evaluated. Ablation was performed once in 27 patients. In 6 patients 2 ablations were performed: In 2 patients 2 lesions were ablated in one session. In 3 patients 2 lesions were ablated in 2 consecutive sessions. In one patient a residual tumor was ablated in a second session. In 2 patients 3 ablations were performed: 1. ablation of 2 lesions in one session, ablation of a third lesion later. 2. ablation of 2 lesions in one session and a residual tumor in a second session.

In most cases (35/45) transarterial lipiodol (Lipiodol Ultra-Fluid, Guerbet GmbH, Sulzbach, Germany) premarking was performed 1 day before the IRE procedure to facilitate delineation of the lesion. In most cases, transarterial superselective sounding of the feeding artery with a 2.1–2.8 French microcatheter was possible. In that position, lipiodol (mean: 2.1 mL; range: 0.5–7 mL) was administered.

All patients were treated with IRE (NanoKnife, Angiodynamics Inc., Latham, NY, USA). General anaesthesia with neuromuscular blockade was necessary to reduce muscle contractions ^13^. Percutaneous needle placement was performed under computed tomography (CT) guidance. In 10 of these cases, a stereotactic navigation system (CAS-One IR, Cascination, Bern, Switzerland) was used to guide the needle placement, primarily if more than three electrodes were used. Electrodes were placed at the edge of the target lesion as parallel as possible and with a target needle distance of 15 to 20 mm ^13^, following the recommendations of the manufacturer. The active needle tip was set at 2 cm in almost all cases (in 3 cases 1.5 cm). After 10 test pulses between each pair of needles, 80 pulses per needle pair with a voltage of 1500 V/cm were applied (pulse length: 90 µs), with a maximum of 3000 V. To achieve a current of 20–35 A, the parameters were adjusted accordingly.

**Figure 1:**
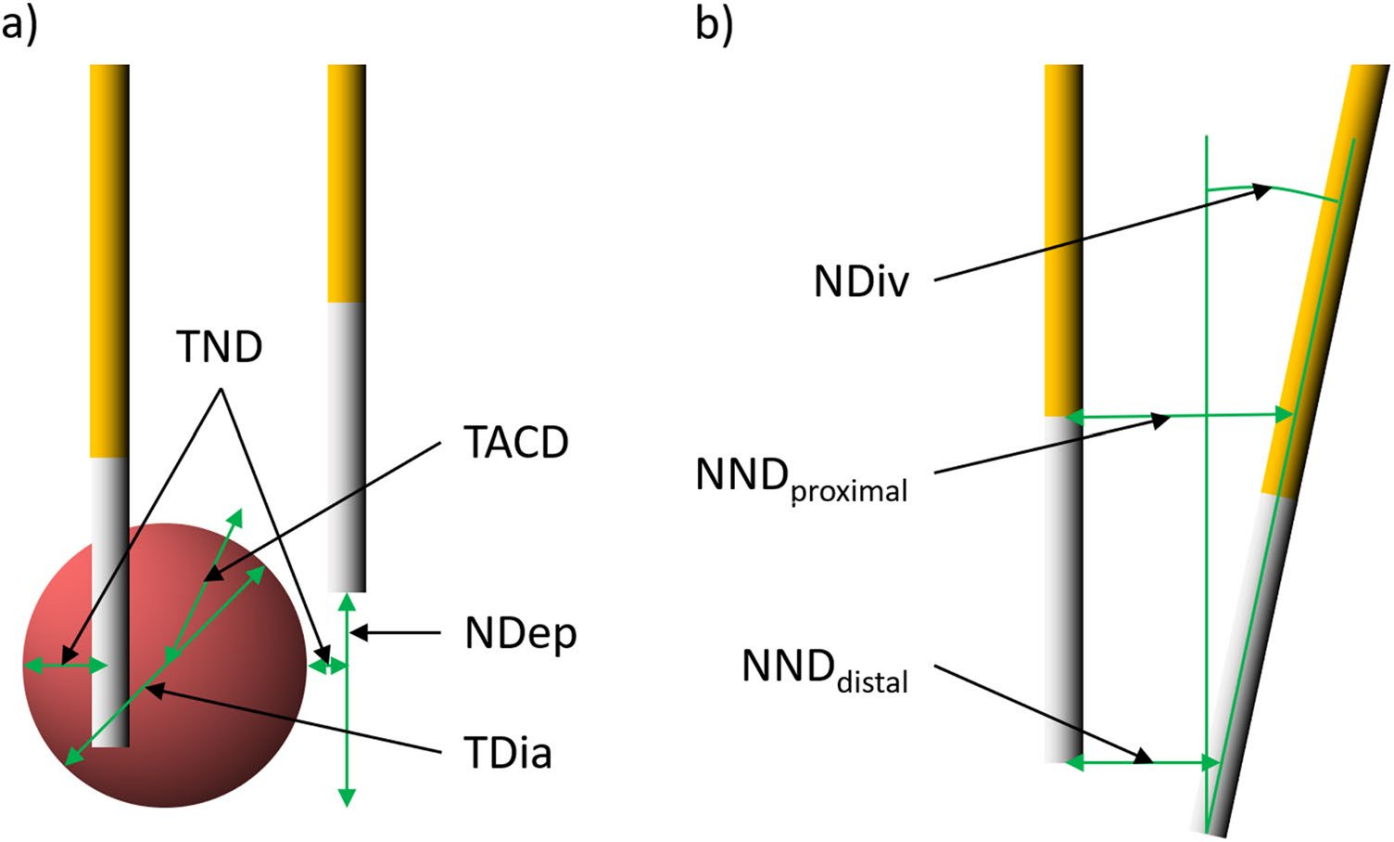
(**a**) Geometric parameters describing the relation of the needles to the lesion and (**b**) the position of the needles in relation to each other. The active needle tip is shown in white; the needle shaft is shown in orange. Please refer to the text for a detailed explanation of the parameters (*TND* tumor-to-needle distance; *TACD* tumor-center-to-ablation-center distance; *NDep* needle depth; *NDiv* needle divergence; *NND* needle-to-needle distance; *TDia* tumor diameter).

Needle geometry was evaluated by measuring the following parameters in each case: Parameters describing needle positioning in relation to the lesion position were tumor-center-to-ablation-center distance (TACD), tumor-to-needle distance (TND), and needle depth (NDep). Parameters describing the position of the needles in relation to each other were needle divergence (NDiv) and needle-to-needle distance (NND; Fig. 1). Measurements of the needle geometry were performed in the CT series immediately before ablation. In most cases lesion visibility was sufficient. If the lesion was not sufficiently visible, correlation with previous contrast enhanced CT series or the pre-interventional MRI to measure lesion-dependent parameters was performed. NND and TND were measured in planes orthogonal to the needle alignment. NDep was measured in planes parallel to the needle orientation. NDep was defined as tumor overlap beyond the most proximal needle tip. To measure TACD, tumor center and ablation center were determined, the distance was measured orthogonal and parallel to the needle orientation. TACD was then calculated as follows:

$$TACD = \sqrt {distance_{parallel}^{2} + distance_{orthogonal}^{2} }$$

The NND was measured distally and proximally. NND_distal_ corresponds to the distance between the needle pairs, measured at the height of the most proximally located needle tip. NND_proximal_, on the other hand, was measured at the height of the end of the selected probe exposure (in almost all cases 2 cm from the probe tip). The needle divergence (NDiv) was calculated from these parameters as follows:

$$NDiv = arctan\left( {\frac{{\left| {NND_{distal} {-} NND_{proximal} } \right|}}{Length\,of\,active\,needle\,tip}} \right)$$

Since a value for NDiv, NND or TND was collected for each needle pair or needle, an average value was calculated for each procedure, which was then included in the statistical calculations. The tumor diameter (TDia) was measured in axial plane. TDia/needle number was calculated in the study to allow a quantification of the needles required in relation to tumor size to achieve a successful ablation. In addition, lesion contact with the liver capsule, portal vein, or liver vein was assessed. Capsule contact was defined as the distance from the lesion border to the capsule of < 2 mm. Contact with the portal vein was assumed if the lesion was adjacent to the left or right main branch (first order) or to its direct branches (second order). Contact with the liver vein was assumed if the lesion was adjacent to a vein with a diameter > 3 mm. All measurements were performed based on a consensus of two readers (with 2 and 12 years of experience) in non-blinded manner.

Descriptive statistics were calculated for collected data, with means and standard deviations determined for normal distributed data and median and interquartile range for nonnormal distributed data. Yate’s chi-squared test or exact chi-squared test were used to compare the categorical data. Continuous data were compared using Student’s *t* test. Receiver-operating characteristic (ROC) curve analysis was performed to determine cutoff values. The significance level for statistical testing was set at *p* < 0.05.

## Ethics approval

This study has been approved by the ethics committee of the medical faculty Heidelberg (Ethikkomission der medizinischen Fakultät Heidelberg, Reference Number: S-442/2019). As the study was retrospective and observational, patient written informed consent was waived by the ethics committee of the medical faculty Heidelberg. All methods carried out were in accordance with the national legal regulations and the ICH-GCP guidelines.

## Results

Clinical parameters such as age, the presence of liver cirrhosis, portal vein hypertension and the Child–pugh score were similar (Table 1). The proportion of male patients was slightly lower in the residual tumor group, but not significant. Tumor diameter and the percentage of ablations with lipiodol premarkings were comparable in both groups as well.

**Table 1:** Baseline characteristics.

	No residual tumor	Residual tumor	*p *value
	Mean ± standard deviation		*t* test
Age (years)	65.7 ± 8.7	62.7 ± 10.9	0.31
Tumor diameter (mm)	17.6 ± 6.0	17.7 ± 7.1	0.97

	Percentage (%)		Yate’s chi-squared test
Sex (male)	80	60	0.28
Chirrosis	93.3	93.3	1
Portal hypertension	69	64.3	1
Child–Pugh A	83.3	80	0.78 (exact chi-squared test)
Child–Pugh B	13.3	20	
Child–Pugh C	3.3	0	
Lipiodol premarking	80	73.3	0.9

**Table 2:** Geometrical parameters measured in both groups. For the abbreviations and visual explanation of the parameters see Fig. 1.

Geometrical parameter	No residual tumor	Residual tumor	*p* value
	Mean ± standard error		*t* test
TACD (mm)	3.2 ± 0.4	11.6 ± 1.4	< 0.001
TND (mm)	1.9 ± 0.3	4.7 ± 1.2	0.04
NDep (mm)	2.1 ± 0.6	6.8 ± 1.5	< 0.01
NDiv (°)	3.7 ± 0.5	7.0 ± 1.2	0.02
Needle number	3.1 ± 0.2	2.4 ± 0.2	0.04
TDia/needle (mm)	5.9 ± 0.3	7.5 ± 0.6	0.01
NND (mm)	15.4 ± 0.7	16.3 ± 0.9	0.4

**Figure 2:**
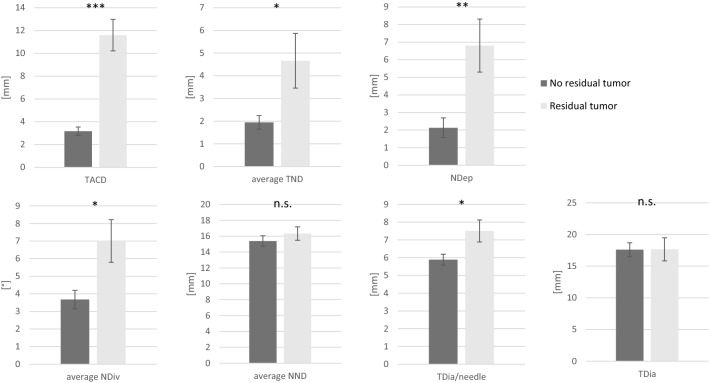
Comparison of the means of the geometrical parameters between patients without residual tumor (n = 30) and with residual tumor (n = 15). Parameters, which describe needle placement relative to the lesion (TACD, TND, NDep), as well as NDiv and TDia/needle, were significantly different. Means ± standard error. *t* tests between both groups. ***p < 0.001, **p < 0.01, *p < 0.05, *n.s.* not significant.

The *t* tests of the measured geometric parameters revealed that correctly positioning the needles near the lesion is crucial for ablation success (Table 2). All parameters describing needle positioning in relation to the lesion location correlated significantly with a successful ablation: TACD and TND were significantly higher in patients with residual tumor (TACD: 11.6 vs. 3.2 mm, *p* < 0.001; TND: 4.7 vs. 1.9 mm, *p* = 0.04; Fig. 2, Table 2). In many cases with residual tumor, NDep was too short (2.1 vs. 6.8 mm tumor overlap beyond the most proximal needle tip, *p* < 0.01). Furthermore, parallel needle placement was important: the mean NDiv was significantly higher in patients with residual tumor (7.0° vs. 3.7°, *p* = 0.02). In addition, a greater number of needles used was significantly correlated with ablation success (3.1 vs. 2.4, *p* = 0.04), and the mean TDia per needle was significantly lower (5.9 vs. 7.5 mm/needle, *p* = 0.01). The mean NND was slightly, but not significantly, lower in patients with residual tumor (15.4 vs. 16.3 mm).

**Figure 3:**
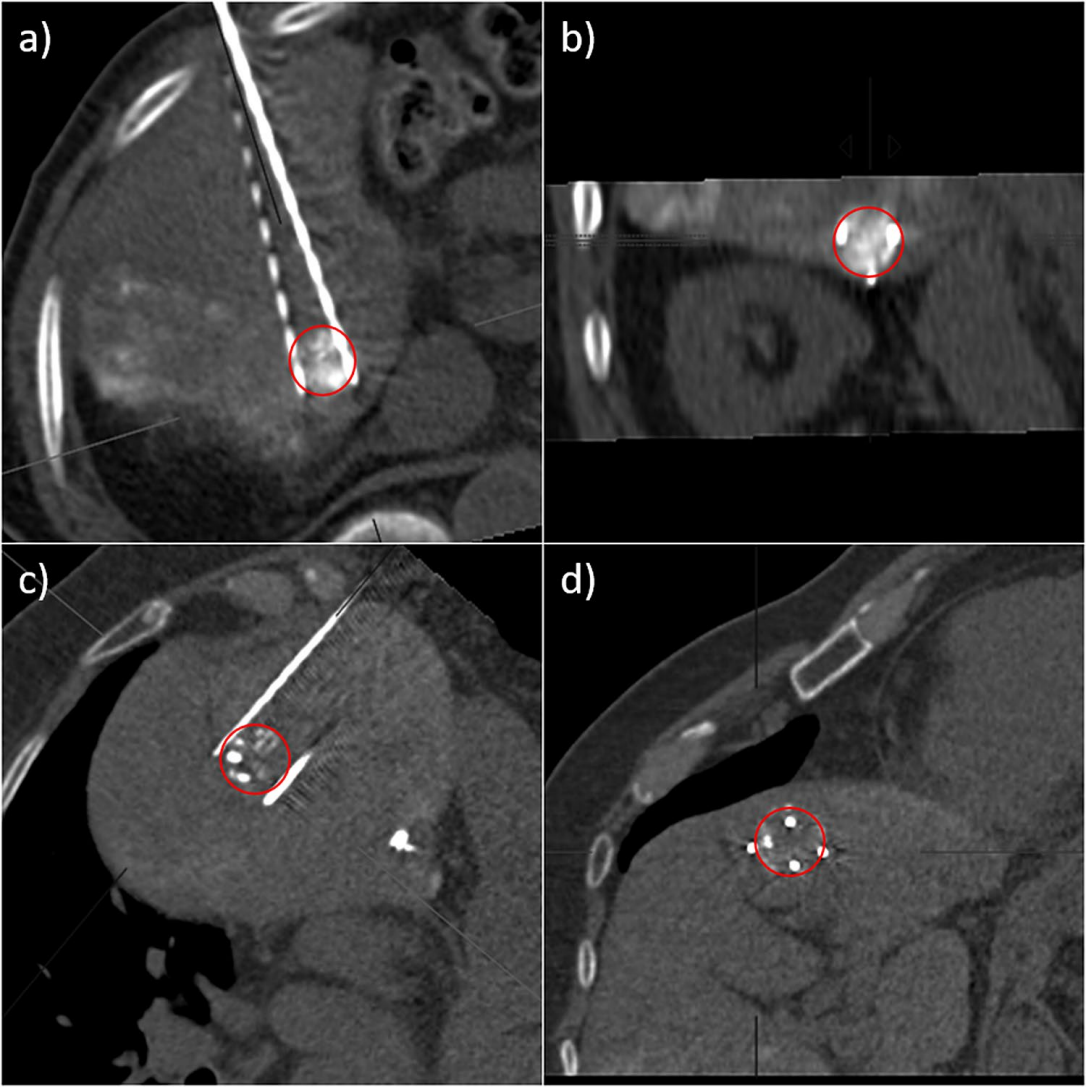
Example of two successful IREs with adequate needle placement. In each case MPRs of the CT with the needle positions immediately before ablation, in parallel alignment to the needle plane (**a**, **c**) and orthogonal to it (**b**, **d**) are shown. Both tumors were marked with Lipiodol the day before, lesions outlined in red. (**a**) and (**b**) Ablation with 3 needles. The three needles were placed adjacent to the tumour with a low tumor-center-to-ablation-center distance (TACD = 1 mm), with sufficient needle depth (Ndep = 0). Ablation was successful, although needle divergence was relatively high (average NDiv = 4.8°). (**c**) and (**d**) Ablation with 4 needles, a stereotactic navigation system was used for needle placement. Here, too, all needles were placed at sufficient depth (NDep = 2 mm) in the area around the edge of the tumor with a sufficiently low tumor-center-to-ablation-center distance (TACD = 5 mm) and almost no needle divergence (average NDiv = 0.7°).

Assistance by the stereotactical navigation system Cas-one IR led to a lower NDiv (2.1° vs. 5.6°, *p* < 0.01). As an example, please note the presented case in Fig. 3c,d, where it was possible to place 4 needles with a very low NDiv using the navigation system. In the residual tumor group, only one IRE was performed with stereotactical navigation system–assistance; however, this did not lead to a significant difference in comparison with the no residual tumor group (percentage of ablations with stereotactic navigation: 6.7% in the group with residual tumors vs. 30% in the group without residual tumors, *p* = 0.16).

**Figure 4:**
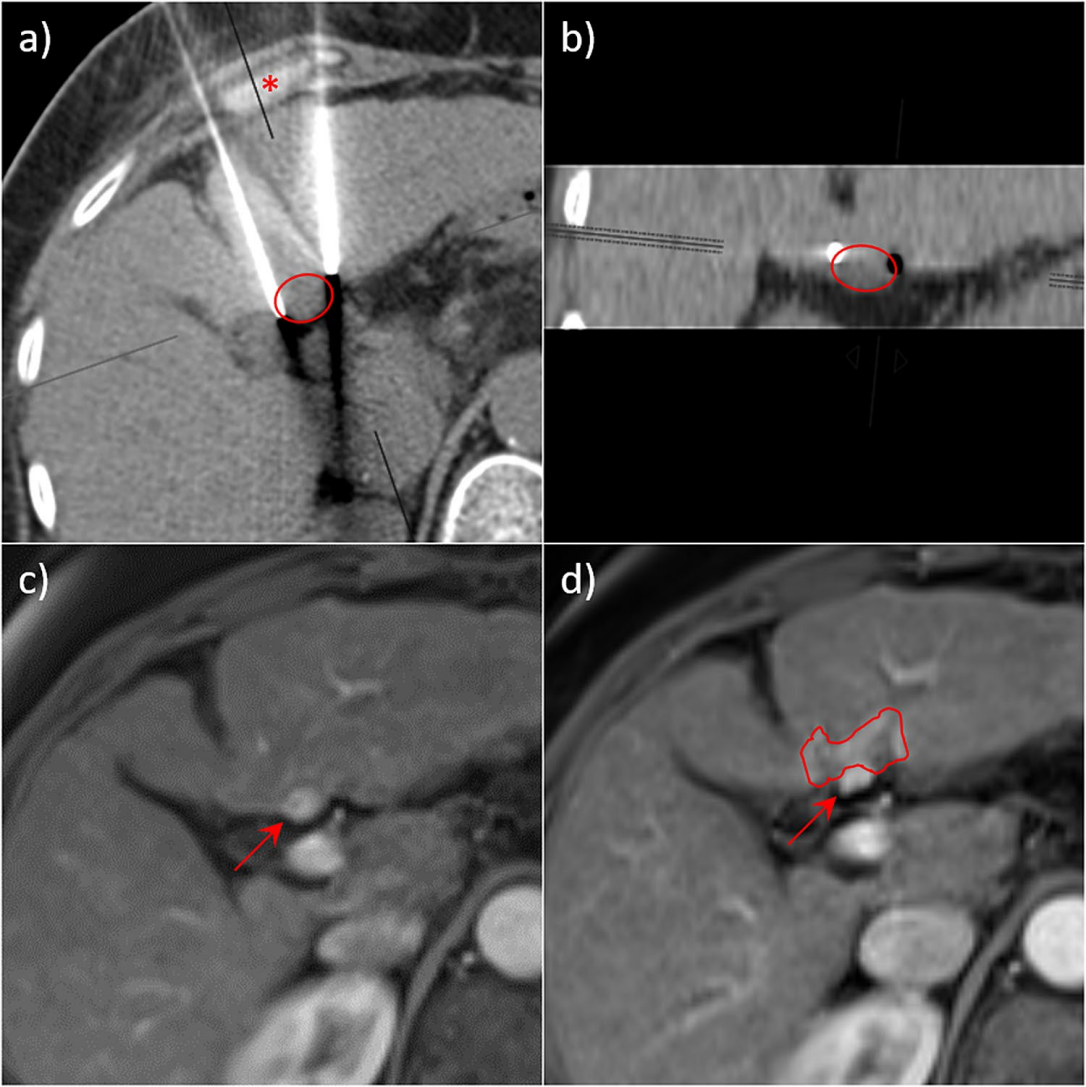
Example of an IRE with residual tumor after ablation with 2 needles. (**a**) and (**b**) show multi-planar reconstructions (MPRs) of the computed tomography (CT) with needle placement immediately before ablation of a hepatocellular carcinoma (HCC), (**a**) in parallel alignment to the needle plane, (**b**) orthogonal to it. The tumor location is outlined as red circle. Here 1. the needle depth was insufficient (Ndep = 11 mm) due to the subcapsular location of the tumor, 2. the needle divergence was high (Ndiv = 8.5°) because of the unfavourable interposition of the rib cartilage (asterix) and 3. the tumor was slightly caudal to the needle plane. This results in a high tumor-center-to-ablation-center distance (TACD = 10 mm). (**c**) Contrast enhanced magnetic resonance imaging (MRI) before ablation showing the hypervascularised HCC (arrow). (**d**) residual tumor 6 weeks after ablation. The distorted ablation area, which spares the tumor area, is outlined in red.

**Figure 5:**
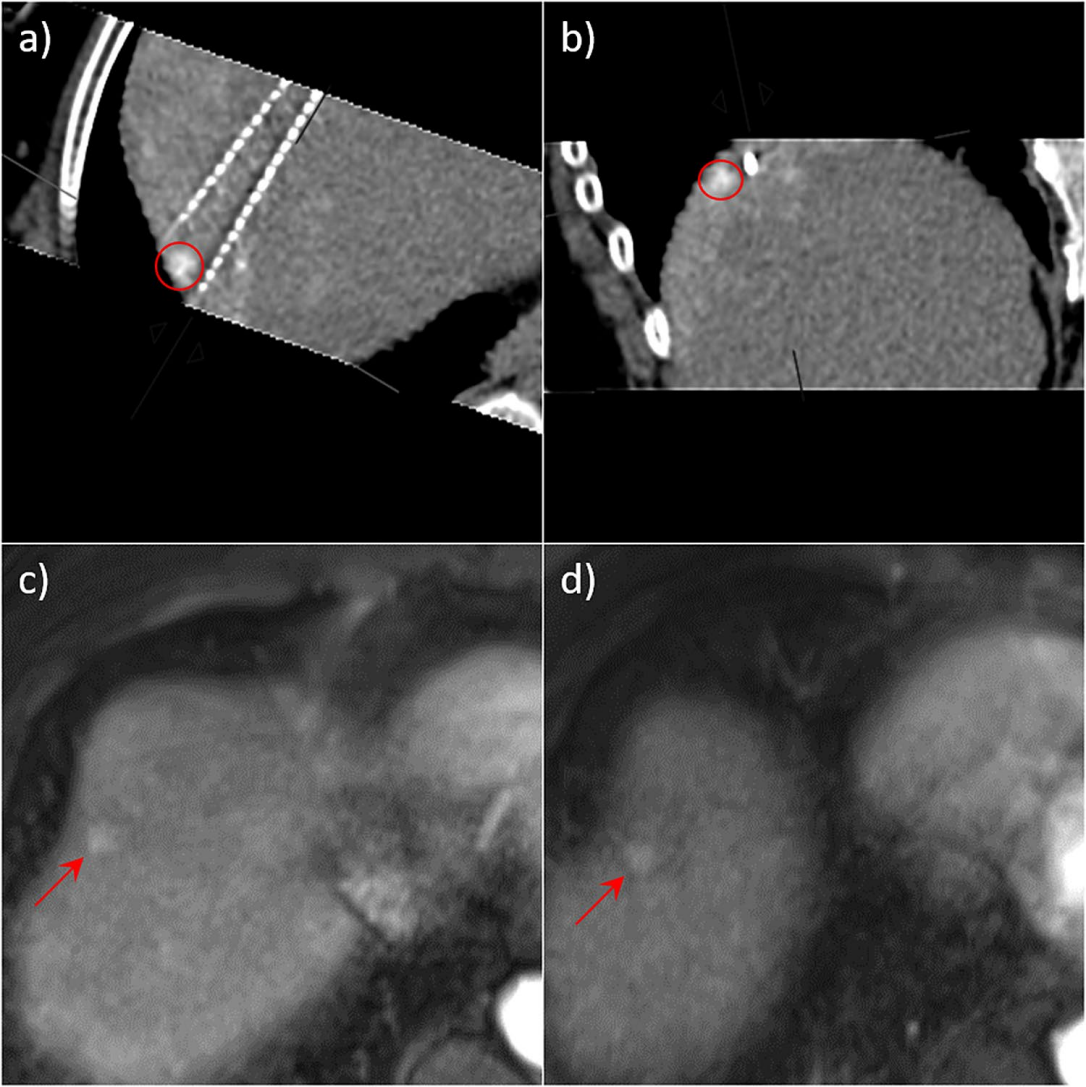
Another example of an IRE with residual tumor after ablation with 2 needles. (**a**) and (**b**) show MPRs of the CT with needle placement immediately before ablation, (**a**) in parallel alignment to the needle plane, (**b**) orthogonal to it. HCC, marked with lipiodol the day before, is outlined as red circle. In this case the depth of the lateral needle was insufficient due to the subcapsular location of the tumor (Ndep = 11 mm), the needle divergence was borderline high (Ndiv = 5.7°) and the tumor-center-to-ablation-center distance was too high (TACD = 8 mm). (**c**) Contrast-enhanced MRI before ablation with hyperarterialised HCC (arrow). (**d**) Residual tumor 6 weeks after ablation.

Subcapsular lesion location (capsule distance < 2 mm) was observed significantly more often in patients with residual tumor (subcapsular location in 11 of 15 cases (73.3%) with residual tumor vs. 9 of 30 cases (30%) without residual tumor, *p* = 0.02, Yate’s chi-squared test). For example, please refer to the two presented ablations in Figs. 4 and 5. In 16 of 30 patients (53.3%) without residual tumor and in 5 of 15 patients (33.3%) with residual tumor, the lesion was in contact with the portal vein (left and right portal vein and its direct branches). In 10 of 30 patients (33.3%) without residual tumor, the lesion was in contact with the liver vein (vessel diameter > 3 mm), compared with 2 of 15 patients (13.3%) with residual tumor.

Binary logistic regression was performed to determine the significant independent predictive factors among TACD, NDiv, and TDia per needle. Among these, only the odds ratio (OR) for TACD was barely significant (OR 5.411, *p* = 0.05).

**Figure 6:**
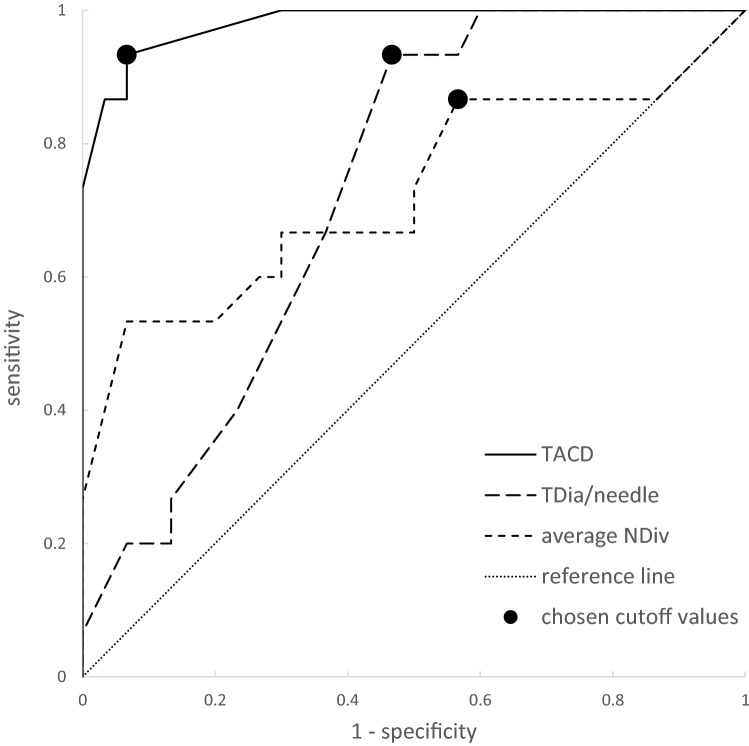
ROC curve analysis calculated for TACD, TDia/needle, and NDiv to determine cutoff values. Chosen cutoff values are indicated as black dots.

The ROC curve analysis was performed to determine cutoff values. As residual tumor should be avoided as much as possible, cutoff values with a high sensitivity but at the cost of specificity were chosen. The analysis showed that the TACD should not be greater than 5.5 mm (sensitivity = 0.93, specificity = 0.93, area under the curve [AUC] = 0.98; Fig. 6). In addition, the TDia per needle should not exceed 5.9 mm (sensitivity = 0.93, specificity = 0.53, AUC = 0.73), and an NDiv greater than 2.4° should be avoided (sensitivity = 0.87, specificity = 0.43, AUC = 0.73) if possible.

The evaluation of each case of residual tumor revealed at least one needle positioning error in all 15 cases. The most common errors were insufficient needle depth and tumor location out of the needle plane (f.e. Figures 4 and 5). Insufficient needle depth was found in 7 of 15 cases (46.7%); subcapsular tumor location was the culprit in 5 of these cases (71.4%). Tumor location out of the needle plane was found in 8 of 15 cases (53.3%), and 2 needle arrangements were especially prone to this problem. Subcapsular tumor location was found in 5 of these cases (62.5%) as well. In 2 of 15 cases (13.3%), the lesion was missed because of poor conspicuity.

## Discussion

The aim of this study was to evaluate reasons for incomplete ablation after IRE in a clinical patient collective and to develop recommendations to avoid this scenario. A special focus was placed on the evaluation of needle geometry, because IRE is a geometrically complex procedure that requires the use of at least two needles. For this purpose, the needle geometry parameters as well as lesion location in a group with incomplete ablations were compared with those of a control group with complete ablations after IRE of HCC.

In all 15 evaluated cases with incomplete ablation, the needle positioning was not optimal. Based on our ROC curve analysis, we determined that the cutoff value of the most significant parameter, TACD, should not exceed 5.5 mm. Other geometrical parameters describing the needle position in relation to the tumor differed significantly as well. Two important reasons for high TACD were found: (1) an insufficient needle depth occurred because of a subcapsular lesion location and (2) in two needle arrangements, the lesion was centered out of the needle plane, so although the needles were placed adjacent to the lesion, an insufficiently ablated tumor area remained. For the latter, an additional ablation needle would have been useful. Correspondingly, the parameter TDia per needle was significantly higher in ablations with residual tumor, which means that in these cases too few needles were used. TDia per needle should not be higher than 5.9 mm based on our ROC curve analysis. This result fits well with the manufacturer's recommendation: 2 needles for tumors up to 1 cm, 3 needles up to 1.5 cm, 4 needles (rectangular arrangement) up to 2 cm and 7 needles for a lesion up to 4 cm in size.

In most of these cases, evaluation of the needle’s position in only axial reconstruction was probably misleading, but multiplanar reconstruction with an evaluation in two planes—parallel and orthogonal to the needle orientation—revealed the mispositioning. The NND was not a problem in our patient collective, because it was in both groups 15–16 mm in average, which is well in line with the manufacturer’s recommendations (i.e., 15–20 mm; ^13^). According to the manufacturer’s recommendation (and ^13^), the needles should be positioned as parallel as possible because of the risk of either overcurrent or ablation gaps. However, a maximum tolerable NDiv was not provided. In the incomplete ablation group, the mean NDiv was significantly higher than in the control group. Based on our ROC curve analysis, the NDiv should not exceed 2.4°. However, this value should be treated with care and is probably set too low (for example see the successful ablation in Fig. 3a,b with an average NDiv = 4.8°), because a high TACD and NDiv were correlated in our groups (Pearson correlation = 0.34, *p* = 0.02), and binary logistic regression including TACD, NDiv, and TDia per needle did not show significance for NDiv.

Previous studies reported that treating tumors > 3 cm ^19^ leads to a higher risk of residual tumor after ablation. Because we complied with this threshold, the maximum TDia in the evaluated collective was 31 mm, and the diameter was 18 mm in both groups. Thus, tumor size has not been a major reason for residual tumors in our center.

Because IRE is a nonthermal ablation method, no heat-sink effect is expected ^20^. Indeed, the proportion of cases with portal vein contact and liver vein contact tended to be higher in the complete ablation group. This result is probably a mirror effect of the finding that the percentage of subcapsular lesion locations was significantly higher in the incomplete ablation group, as subcapsular lesions typically have no contact with large vessels. Subcapsular lesion location was, in our opinion, not an independent risk factor for incomplete ablation (logistic regression including TACD, capsule contact, and NDiv showed significance only for the OR of TACD: OR = 3.4, *p* = 0.015), but it did lead to needle misplacement, as described above.

In a prospective study ^9^ as well as in a retrospective one ^14^, stereotactical navigation systems (CAS-One IR) were shown to reduce procedure length and increase the accuracy of IRE, measured as needle deviation. Here, 10 of the evaluated cases were performed under stereotactical assistance, with only one case showing incomplete ablation. In our study, needle deviation was significantly lower when using stereotactic navigation, too (NDiv = 2.1° vs. 5.6°, *p* < 0.01).

All ablations were performed with an applied voltage of 1500 V/cm for 80 pulses with an aspired current of 20 to 35 A; during successful IRE, the current should increase ^21^. However, we did not evaluate these data in detail in this study. So inhomogeneous tissue resistance might be another possible reason for the incomplete ablation in our cases. Though, a relevant effect should not have arisen in this study. First, because the proportion of patients with liver cirrhosis and the Child–Pugh score were similar in both groups (Table 1). Second, in a porcine model, IRE liver destruction was shown to be equally effective in cirrhotic and noncirrhotic liver ^22^.

One major limitation of this study is that the evaluation of reasons for incomplete ablation was based on only 15 residual tumor cases, so the differentiation of potential statistical independent factors for incomplete ablation was difficult.

Another limitation is that this was only a single-center experience, and results may not apply to other centers, because needle placement is operator dependent. Because of the study’s retrospective design, it was not possible to systematically vary the needle placement in order to carefully differentiate the different factors for incomplete ablation. In addition, differences in complication rates between groups were not evaluated (e.g., whether vessels were intact after the intervention).

A potential investigator bias is possible since the measurements were not done in a blinded manner. Measurements that only include needle-to-needle positions should be subject to a relatively small error. For the calculation of the needle divergence, the needles were considered to lie approximately in one plane. However, given the overall relatively small needle divergences (the maximal calculated needle divergence was 16.7°), the resulting calculation error should be negligible. Measurements in which the needle position was determined in relation to the tumor were more difficult because the tumor was not clearly visible in some cases. In these cases, the tumor position had to be correlated and projected using other contrast enhanced series or previous MRI pictures.

In summary, this is, to our knowledge, the first clinical study in which needle placement errors were examined in detail. We showed that, at least in our cases, placement errors were the main cause of incomplete ablation, with TACD as the most important geometric parameter. Careful evaluation of needle positioning in at least two multiplanar reconstructions, parallel and orthogonal to the needle orientation, as well as a very parallel needle placement can probably avoid most residual tumors. Stereotactical navigation systems might help to ensure correct needle positioning. Further studies with a multicentric evaluation and a larger case number would be helpful to confirm these recommendations. In addition, studies evaluating electrical current patterns during ablation are needed, as this is another possible reason for residual tumors after ablation, which was not evaluated in detail in this study.

## References

[1] Lu DS, Yu NC, Raman SS, Limanond P, Lassman C, Murray K, Tong MJ, Amado RG, Busuttil RW (2005). Radiofrequency ablation of hepatocellular carcinoma: treatment success as defined by histologic examination of the explanted liver. Radiology.

[2] Nault JC, Sutter O, Nahon P, Ganne-Carrié N, Séror O (2018). Percutaneous treatment of hepatocellular carcinoma: state of the art and innovations. J. Hepatol..

[3] Davalos RV, Mir IL, Rubinsky B (2005). Tissue ablation with irreversible electroporation. Ann. Biomed. Eng..

[4] Vroomen L, Petre EN, Cornelis FH, Solomon SB, Srimathveeravalli G (2017). Irreversible electroporation and thermal ablation of tumors in the liver, lung, kidney and bone: What are the differences?. Diagn. Interv. Imaging.

[5] Jiang C, Davalos RV, Bischof JC (2015). A review of basic to clinical studies of irreversible electroporation therapy. IEEE Trans. Biomed. Eng..

[6] Ben-David E, Ahmed M, Faroja M, Moussa M, Wandel A, Sosna J, Appelbaum L, Nissenbaum I, Goldberg SN (2013). Irreversible electroporation: treatment effect is susceptible to local environment and tissue properties. Radiology.

[7] Silk M, Tahour D, Srimathveeravalli G, Solomon SB, Thornton RH (2014). The state of irreversible electroporation in interventional oncology. Semin. Interv. Radiol..

[8] Rubinsky B, Onik G, Mikus P (2007). Irreversible electroporation: a new ablation modality–clinical implications. Technol. Cancer Res. Treat..

[9] Beyer LP, Pregler B, Nießen C, Schicho A, Haimerl M, Jung EM, Stroszczynski C, Wiggermann P (2016). Stereotactically-navigated percutaneous irreversible electroporation (IRE) compared to conventional IRE: a prospective trial. PeerJ.

[10] Edd JF, Davalos RV (2007). Mathematical modeling of irreversible electroporation for treatment planning. Technol. Cancer. Res. Treat..

[11] Sweeney DC, Robert EN, Rafael VD, Meijerink MR, Scheffer HJ, Narayanan G (2018). Multi-scale Biophysical Principles in Clinical Irreversible Electroporation. Irreversible Electroporation in Clinical Practice.

[12] Martin RC (2015). Irreversible electroporation of locally advanced pancreatic neck/body adenocarcinoma. J. Gastrointest. Oncol..

[13] Narayanan G (2015). Irreversible electroporation. Semin. Interv. Radiol..

[14] Beyer LP, Pregler B, Michalik K, Niessen C, Dollinger M, Müller M, Schlitt HJ, Stroszczynski C, Wiggermann P (2017). Evaluation of a robotic system for irreversible electroporation (IRE) of malignant liver tumors: initial results. Int. J. Comput. Assist. Radiol. Surg..

[15] Kalra N, Gupta P, Gorsi U, Bhujade H, Chaluvashetty SB, Duseja A, Singh V, Dhiman RK, Chawla YK, Khandelwal N (2019). Irreversible electroporation for unresectable hepatocellular carcinoma: initial experience. Cardiovasc. Interv. Radiol..

[16] Mafeld S, Wong JJ, Kibriya N, Stenberg B, Manas D, Bassett P, Aslam T, Evans J, Littler P (2019). Percutaneous irreversible electroporation (IRE) of hepatic malignancy: a bi-institutional analysis of safety and outcomes. Cardiovasc. Interv. Radiol..

[17] Eller A, Schmid A, Schmidt J, May M, Brand M, Saake M, Uder M, Lell M (2015). Local control of perivascular malignant liver lesions using percutaneous irreversible electroporation: initial experiences. Cardiovasc. Interv. Radiol..

[18] Tian G, Zhao Q, Chen F, Jiang T, Wang W (2017). Ablation of hepatic malignant tumors with irreversible electroporation: a systematic review and meta-analysis of outcomes. Oncotarget.

[19] Zimmerman A, Grand D, Charpentier KP (2017). Irreversible electroporation of hepatocellular carcinoma: patient selection and perspectives. J. Hepatocell Carcinoma.

[20] Charpentier KP, Wolf F, Noble L, Winn B, Resnick M, Dupuy DE (2011). Irreversible electroporation of the liver and liver hilum in swine. HPB.

[21] Beitel-White N, Bhonsle S, Martin R, Davalos RV (2018). Electrical Characterization of Human Biological Tissue for Irreversible Electroporation Treatments. Conf. Proc. IEEE. Eng. Med. Biol. Soc..

[22] Abdelsalam ME, Chetta JA, Harmoush S, Ensor J, Baugh A, Dixon K, McWatters A, Wallace MJ, Tam A, Avritscher R (2013). Irreversible electroporation (IRE) in cirrhotic liver: preliminary experience in a large animal model. J. Vasc. Interv. Radiol..

